# Matrix for Mucosal Regeneration in Transoral Glossectomy for Squamous Cell Carcinoma: Objective and Subjective Functional Evaluation

**DOI:** 10.3390/curroncol30020104

**Published:** 2023-01-17

**Authors:** Alberto Deganello, Paolo Bosio, Lorenzo Giannini, Federico Parolini, Giulia Berretti, Alessandra Sordi, Vittorio Rampinelli, Tommaso Gualtieri

**Affiliations:** 1 Otolaryngology Head and Neck Surgery Department of IRCCS, National Cancer Institute (INT), 20133 Milan, Italy; 2 Otolaryngology Head and Neck Surgery Department, Spedali Civili di Brescia, 25123 Brescia, Italy

**Keywords:** partial glossectomy, oral cancer, transoral surgery, dermal matrix, oral reconstruction

## Abstract

Background: Numerous options to manage local reconstruction following transoral partial glossectomy are possible. In this work, we present our experience using a matrix for mucosal regeneration, Integra^®^, after transoral resections of squamous cell carcinoma of the oral tongue. Methods: A retrospective analysis of patients treated for tongue carcinoma and reconstruction with Integra^®^, from September 2017 to September 2022. Functional outcomes were evaluated by measuring swallowing and speech abilities, tongue motility, and subjective quality of life. Results: The series accounts for 13 consecutive patients, staged from Tis to T3, no positive resection margins were found, average defect size was 17.8 cm^2^. The average histologically measured depth of invasion was 4.1 mm (range 2–12 mm), and no recurrences were observed during follow-up. All patients maintained excellent swallowing function, the average number of recognized words by an external listener during a phone call was 70.5 out of 75, the lingual motility test was good (a mean score of 4.5 out of 6 movements correctly executed) and subjective questionnaires results were optimal. Less satisfying functional results were recorded in elderly patients receiving a wider surgical resection. Conclusions: This reconstructive technique for allows obtaining optimal healing and functional outcomes in patients with tumors suitable for transoral glossectomy.

## 1. Introduction

Reconstruction of head and neck defects requires a thoughtful approach in order to maximize the restoration of form and function. Free flap transposition represents the gold standard for head and neck reconstruction, since every requirement in terms of restoration of support, cover, and the lining is met by the transposition of fasciocutaneous, musculocutaneous, osseous, osteocutaneous, or chimeric free flaps [1,2]. The reconstructive requirements in case of defects resulting from transoral glossectomy for squamous cell carcinoma (SCC) are limited to provide timed healing and adequate resurfacing, preventing excessive scar retraction, that eventually will cause functional impairments. In fact, when surgery does not create communication between different compartments (i.e., oral cavity and neck spaces), the transposition of a flap restoring the separation is not strictly necessary. In this light, the amount of resected tongue tissue should not be considered the mainstay factor for requiring a flap transposition, but rather the characteristics of the defect. Mobile tongue defects, with at most minimal extension to the floor of the mouth, can be managed without the need for a flap. For these reasons, we explored the possibility of using dermo–epidermal regeneration matrices, originally used in plastic surgery for head and neck reconstruction for severe burns, trauma, surgical wounds, and skin cancer [3,4]. In this work, we present our preliminary experience using the Integra^®^ matrix (Plainsboro, NJ, USA) for mucosal regeneration, after transoral resection of squamous cell carcinoma. Integra^®^ is a bilayer matrix formed by an inner porous layer made of cross-linked bovine tendon type I collagen and glycosaminoglycan, and an outer layer made of a thin non-resorbable, semi-permeable polysiloxane (silicone sheet), that proved to be effective for intraoral resurfacing [5].

## 2. Materials and Methods

This study is a retrospective analysis on patients treated with curative intent for tongue carcinoma and reconstruction with Integra^®^, at the Otolaryngology Head and Neck Surgery Department of IRCCS National Cancer Institute (INT), Milan, and of the University of Brescia, Italy, from September 2017 to September 2022, by the first Author (AD).

Inclusion criteria were adult patients (>18 years old) receiving transoral glossectomy with Integra^®^ bilayer wound matrix reconstruction; cTis-3 N0 SCC of the oral tongue [6]. Exclusion criteria were non-SCC histology; clinically evident or suspect lymph node metastasis at diagnosis; defects resulting from pull-through resection; the presence of second primary simultaneous head and neck cancers; previous treatment for head and neck SCC; distant metastasis at the time of surgery.

**Figure 1:**
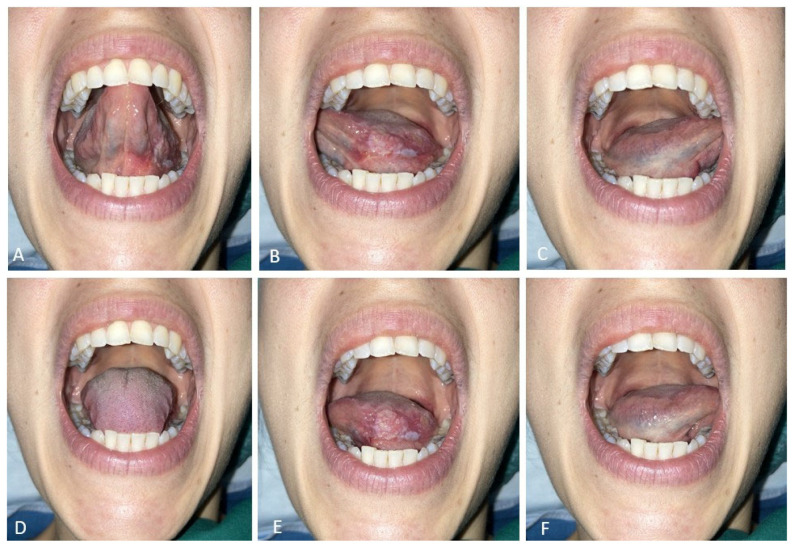
Preoperative tongue mobility test. (**A**–**C**): upper movements; (**D**–**F**): lower movements.

**Figure 2:**
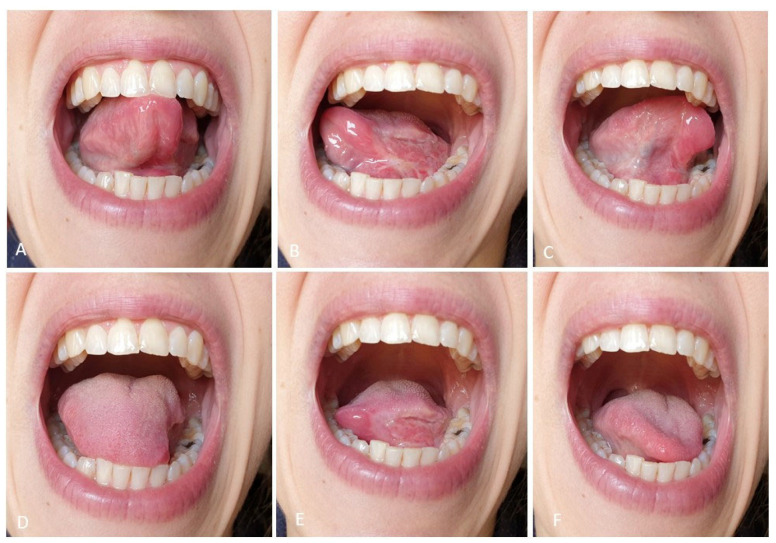
Same patient, postoperative mobility test. (**A**–**C**): upper movements; (**D**–**F**): lower movements.

Pre-operative (Figure 1), and post-operative (Figure 2) tongue motility was evaluated using a newly defined test, introduced by this work. 

All patients were asked to perform six tongue tasks keeping the mouth wide open. These were judged by the examiner as score 1, correct movement; score 0, incorrect or not possible movement. With the tip of the tongue, patients tried to reach the palatal surface of the upper incisors; the palatal surface of the upper right molar region; the palatal surface of the upper left molar region; the lingual surface of the lower incisors; the lingual surface of the lower right molar region; the lingual surface of the lower left molar region. These movements were divided into two subgroups, superior lingual motility (first three movements), inferior lingual motility (last three movements).

**Figure 3:**
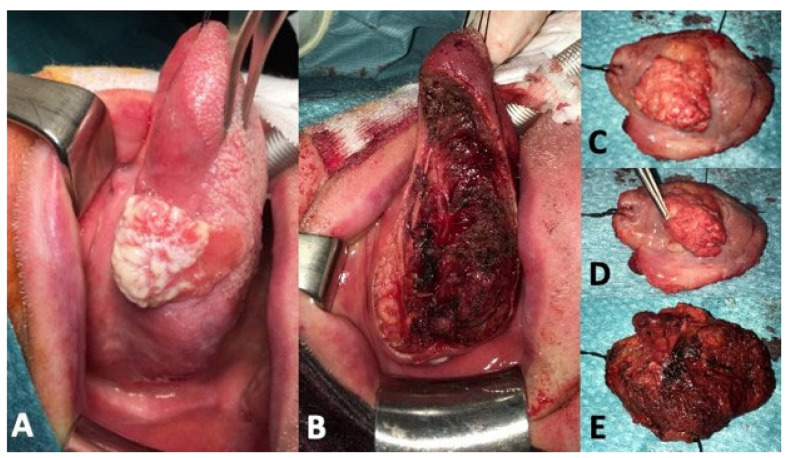
Tumor resection. (**A**): cT2N0 at the middle third of the left lingual margin. (**B**): surgical defect after transoral resection. (**C**–**E**): specimen appearance.

Surgical technique and post-operative care were standardized. Surgery started by marking the mucosal limits of the tumor and then gaining adequate mucosal margins of at least 15 mm in all directions. The resection first started gaining approximately 1 cm of depth at the posterior margin, then it followed an anterior to posterior direction with constant careful palpation ensuring adequate centimetric deep margin (Figure 3). In our opinion, the identification and ligation of the main trunk of the lingual artery, at the posterior aspect of the resection, is crucial to avoid temporary tracheostomy, preventing catastrophic postoperative bleeding [7]. Tumors with a clinically estimated depth of invasion (DOI) >2 mm also received a selective neck dissection of levels I-III. 

**Figure 4:**
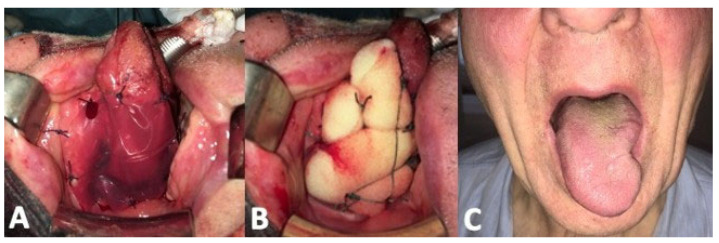
Integra^®^ reconstruction. (**A**): immediate intraoperative placement. (**B**): surgical sponge placement. (**C**): postoperative tongue protrusion.

Integra^®^ was sutured around the tongue defect placing the matrix in close contact with the raw muscular surface. In order to exert constant pressure preventing detachment or fluid collection, a sponge previously soaked with antibiotic solution and then squeezed was sutured against the silicone layer. We used Clindamycin, but whatever antibiotic which prevents bacterial proliferation within the sponge is appropriate. The sponge was then removed 5 days after surgery, while the silicone layer was removed on postoperative day 15 (Figure 4). 

The raw surface of the defect was measured in all patients immediately after resection, by comparing it with the dimensions of the Integra matrix, which measures 5 × 4 cm, so providing a surface of 20 cm^2^. 

An assessment of post-operative pain was made with the Visual Analogue Scale (VAS).

At the 3-month follow-up, patients were submitted to objective tests such as video nasal endoscopic evaluation for swallowing assessment (VEES), a phone call for an evaluation of speech articulation, and a tongue motility test. Furthermore, the final dimensions of the healed and re-epithelized defects were measured and compared with the initial resection area, to estimate the amount of shrinkage and wound retraction. 

VEES was performed with a semiliquid bolus and coupled with a trans-nasal evaluation of pharyngeal post-swallowing pooling and bolus inhalation/penetration. The outcomes were defined according to the three-point scale proposed by Donzelli et al. [8]: score 1, no laryngeal vestibule food entering; score 2, laryngeal vestibule food entering without penetration or aspiration; score 3, tracheal aspiration. 

To evaluate speech articulation, during a phone call, patients were asked to read a list of 75 words containing the most resection-influenced phonemes to an inexpert listener. Edentulate patients read the wordlist wearing their dental prosthesis. A score from 0 to 5 points was then assigned, according to a scale proposed by Grammatica et al. [9], depending on the number of words recognized for each phoneme.

Subjective evaluation of swallowing, speech ability, and quality of life were also measured in all patients using the EORTC H&N35, and UWQOL v.4 questionnaires. 

All data were collected in a database and a non-inferential analysis was conducted.

## 3. Results

### 3.1. Clinical Results

The series accounts for 13 patients, 7 men and 6 women, with a mean age of 58.2 years old. In one case, the resection was extended to the floor of the mouth (Figure 3). Functional assessments were performed 3 months after surgery. Pathological staging revealed three carcinomas in situ, 7 pT1, 2 pT2, and 1 pT3, grading was G2 for 6 patients and G1 for four patients. The average size of the surgical defect was 17.8 cm^2^ (range 12–22 cm^2^), with a definitive re-epithelization surface at 3 months of 10.3 cm^2^.

Selective neck dissection of levels I-II-III was performed in eight cases, and pN0 histological status was always confirmed. Wide resection margins were obtained in 10 patients and close (from 2 to 5 mm) in three. No positive margins were found. Perineural invasion (PnI) was found in five patients and lymphovascular invasion (LvI) in one. The average pathological DOI was 4.1 mm (range 2–12 mm). None of the patients received postoperative adjuvant treatment. For the first four patients of the series, a nasogastric feeding tube was intraoperatively placed and maintained for the first 5 postoperative days and removed with the sponge, while the following nine patients resumed immediate oral feeding from the first postoperative day, despite the presence of the sponge, which did not interfere with swallowing.

The tumors were located at the anterior third of the tongue in six patients, at the medium third in the remaining seven. At the time of removal of the silicone layer, the biological matrix was effectively resurfacing the entire defect. No patient had experimented recurrence of the disease at the time of functional evaluation, and all patients remained free of disease during further routine follow-up consultations. The recorded postoperative VAS pain score was 1.8 (range 0–4).

A summary of clinical results is presented in Table 1.

### 3.2. Functional Results

VEES. A Donzelli score 1 was observed in all cases, and minimal pharyngeal post-swallowing pooling was observed in two patients. 

Speech evaluation. The mean number of words correctly recognized by an inexpert listener during a phone call was 70.5 (range 60–75).

Tongue motility test. The average score was 4.5 out of 6, with 2.67 out of 3 for the superior lingual movements and 1.83 out of 3 for the inferior ones. 

Subjective test evaluation. Regarding swallowing function: the mean EORTC H&N35 and UWQOL v.4 scores were >80% in all patients. For speech articulation: the mean EORTC H&N35 score was: 60% to 80% in three patients and above 80% in the remaining 10 patients; the UWQOL v.4 score was: 60% to 80% in four cases and above 80% in the remaining eight patients. Less satisfying functional results were recorded in elderly patients receiving a wider surgical resection. 

A summary of functional results is presented in Table 2.

## 4. Discussion

In the treatment of oral cancer, the reconstruction may be challenging, and the solution must be tailored to the defect and patient characteristics [10,11,12]. Primary closure of intraoral defects, and similarly, healing by secondary intention, is often known to cause significant scar retractions, and residual tongue fixation, with a potential impact on functional outcomes. In the case of transoral resections, the transposition of a flap will provide volume and resurfacing [1,11,12]. However, the reconstructive flap is a static tissue replacing a dynamic one, with the residual tongue remaining the only functioning tissue. In this light, the amount of resected oral tongue should not be considered the mainstay factor for requiring or not a flap transposition, but rather the advantages and disadvantages of placing a static burden on the residual tongue versus a secondary resurfacing and, moreover, the characteristics of the defect (communication with the neck, extension to the floor of the mouth, to the mandible, to the base of the tongue). Therefore, for intraoral defects that do not mandate a regional or free flap, re-epithelization matrices might gain widespread application, overcoming the disadvantages of skin grafts, including donor site morbidities, such as the risk of infection, scarring, patient discomfort [13,14,15]. A recent systematic review reported the safe use of human, porcine and bovine matrices in a wide spectrum of clinical applications [16]. The application of these tools after partial glossectomy combines the possibility of limiting scar retraction, covering the defect, decreasing postoperative pain, and discomfort, with the absence of additional wounds (graft donor site) on the patient, leading to ultimate re-epithelization by oral mucosa [5]. 

Although the costs of a dermal regeneration matrix are not negligible, reduced operative and hospitalization times have been shown to offset the initial expenses [5,15]. In our series, every integra sheet costs around 800 Euros. We did not perform a cost analysis; however, in our opinion, Integra^®^ reconstruction is valuable: we stopped using nasogastric feeding, the tracheostomy was avoided in all cases, and the global comfort of the patients was very high.

Our work shows how this surgical technique is safe and effective in early oral cancers and in selected locally advanced oral cancers (<T4), localized at the level of the mobile tongue with possibly minimal extension to the oral floor. The Integra^®^ matrix proved to promote re-epithelization of the defect with newly formed mucosa, canalizing second intention wound healing, preventing the formation of excessive retracting scars, with a resulting resurfaced area measuring approximately 60% of the initial surgical bed. The postoperative comfort of all patients was testified by the recorded pain scores, which were negligible in all cases. Moreover, meticulous resection and the routine ligation of the lingual artery during surgery, together with appropriate compression and fixation provided by the sponge, allowed the omission of the tracheostomy in all cases, further favouring the postoperative course.

Our main target was the functional evaluation using objective and subjective tests. As can be seen from the results, functional outcomes were optimal in all patients. In fact, all our patients have had no swallowing problems (Donzelli level 1), and only two of them presented some pharyngeal food residues at VEES, without any impact on daily life. 

In relation to speech intelligibility, we observed optimal results, with a mean of 70.5 of 75 words recognized during a phone call. According to a study proposed by Chang et al. [17] that defined the word intelligibility of >80% as score 4, we obtained an ultimate intelligibility in 84.6% of the patients (*n* = 11). Analyzing the patients who reported a score <80%, we found that they were elderly (73 and 78) with wide surgical defects (21 cm^2^ and 22 cm^2^).

Regarding tongue motility, we observed optimal results (higher than the average score obtained of 2.67 out of 3) in 66.7% of patients (*n* = 8). Patients reporting lower scores (*n* = 4) were elderly with large defects.

Finally, the optimal clinical and functional results are reflected by the questionnaire results.

Due to the small sample size, it was not possible to perform an inferential statistical analysis. Our study also lacks a control group for results comparison. A cohort of transoral resections with skin graft reconstruction could be the ideal match. Nevertheless, we explored the use of Integra^®^ because in our experience we were disappointed by the troublesome postoperative course following skin graft resurfacing of oral defects. We faced frequent graft necrosis and local infections, and we moved away from this solution.

These preliminary results encourage the continuation of treatment of selected tongue carcinomas with this surgical and reconstructive technique. Of paramount importance in this sense is the careful selection of the cases. Dimensions and location of the tumor are of paramount importance. The surgeon must be able to surround the tumor in all directions by palpation and still have at least 15 mm of normal mucosa/muscle beyond the visible and palpatory tumor edges; this will set the resection margins. Tumors located at the posterior third of the oral tongue and tumors extending to the floor of the mouth will be better addressed with a pull-through approach, since respectively the transoral control of the posterior extension is troublesome when the intended margin must fall behind the pharyngo-lingual fold, and the deep margin must encompass the mylohyoid muscle if the tumor extends to the floor of the mouth. Another important aspect is that the transoral resection does not remove the T-N tract; therefore, tumors presenting with evident or clinically suspect neck nodes are better addressed with a pull-through approach ensuring the removal of the primary tumor together with the T-N tract and neck lymph nodes. 

In this light, T1-3 lesions arising from the anterior two-thirds of the tongue, without clinically suspect lymph nodes are ideal candidates. If positive neck nodes are eventually found at histopathologic evaluation, adjuvant therapy is warranted.

Another important aspect that should be considered is that MRI may overestimate the DOI [18,19] because of peritumoral edema at the infiltrative front, as well as in exophytic tumors. Tongue US DOI evaluation proved to be more accurate [20]. The clinician must be aware of these aspects since a thoughtful preoperative staging could increase the number of cases suitable for a transoral resection with Integra^®^ reconstruction sparing unnecessary flap transposition. Another advantage of this reconstructive technique versus a reconstruction with flaps is the possibility of relying on a prompt detection of a local recurrence. In fact, the surgical bed remains in open view, easily inspectable during follow-up, either by visual assessment, and palpation, and it is more difficult for a local recurrence to grow clinically undetected because it cannot be buried under the flap.

Taken all together, we think that reconstruction with Integra^®^ is not indicated in large buccal mucosa resections involving the buccinator muscle, or when the resection extends far beyond the oral tongue encompassing the alveolar ridge, the oropharynx, the buccal mucosa, or when large denuded bony areas must be covered. In addition, when a subtotal/total glossectomy is required, the final lingual volume achieved by flap reconstruction (free or pedicled) allows for better functional performance, in terms of swallowing and phonation management.

## 5. Conclusions

In conclusion, we can affirm that the reconstructive method analyzed in our study (an off-label use of Integra^®^ matrix) allows for obtaining optimal functional outcomes in patients with intraoral surgical defects of limited size. Less favorable outcomes were obtained in two elderly patients receiving large resections. The limitations of our study are mainly related to the small cohort and lack of a control group.

## Figures and Tables

**Table 1:** Surgical and pathological characteristics of the tumors (1,2 Perineural and Lymphovascular Invasion).

	pT	Margins	PnI ^1^	LvI ^2^	Subsite	Initial Defect Size (cm^2^)	Final Defect Size (cm^2^)	Non-Evidence of Disease (Months)
Pt.1	2	Close	+	-	Ant	20	12	60
Pt.2	3	Negative	+		Middle	22	13	56
Pt.3	1	Negative	-	-	Ant	19	9	47
Pt.4	1	Close	+	+	Middle	15	8	40
Pt.5	1	Close	-	-	Middle	18	10	33
Pt.6	Tis	Negative	//	//	Ant	15	7	29
Pt.7	1	Negative	-	-	Ant	17	9	23
Pt.8	1	Negative	-	-	Middle	18	11	18
Pt.9	Tis	Negative	//	//	Middle	12	7	15
Pt.10	1	Negative	+	-	Ant	18	11	9
Pt.11	Tis	Negative	//	//	Middle	16	10	7
Pt.12	1	Negative	+	-	Middle	21	13	4
Pt.13	2	Negative	//	//	Ant	20	14	3

+ positive, - negative, // not reported.

**Table 2:** Functional tests.

Test		Results
Donzelli Score	I	100%
	II	0%
	III	0%
Post-Swallowing pooling	Yes	16.7%
	No	83.3%
Speech Evaluation	Average recognized words	70.5/75
Lingual Motility Score	Superior	2.67/3
	Inferior	1.83/3

## Data Availability

Data are contained within the article.
